# Evaluation of optimal methods and ancestries for calculating polygenic risk scores in East Asian population

**DOI:** 10.1038/s41598-023-45859-w

**Published:** 2023-11-06

**Authors:** Dong Jun Kim, Joon Ho Kang, Ji-Woong Kim, Myeong Jae Cheon, Sun bin Kim, Young Kee Lee, Byung-Chul Lee

**Affiliations:** Genoplan Korea, Seoul, Korea

**Keywords:** Genetic predisposition to disease, Genotype

## Abstract

Polygenic risk scores (PRSs) have been studied for predicting human diseases, and various methods for PRS calculation have been developed. Most PRS studies to date have focused on European ancestry, and the performance of PRS has not been sufficiently assessed in East Asia. Herein, we evaluated the predictive performance of PRSs for East Asian populations under various conditions. Simulation studies using data from the Korean cohort, Health Examinees (HEXA), demonstrated that SBayesRC and PRS-CS outperformed other PRS methods (lassosum, LDpred-funct, and PRSice) in high fixed heritability (0.3 and 0.7). In addition, we generated PRSs using real-world data from HEXA for ten diseases: asthma, breast cancer, cataract, coronary artery disease, gastric cancer, glaucoma, hyperthyroidism, hypothyroidism, osteoporosis, and type 2 diabetes (T2D). We utilized the five previous PRS methods and genome-wide association study (GWAS) data from two biobank-scale datasets [European (UK Biobank) and East Asian (BioBank Japan) ancestry]. Additionally, we employed PRS-CSx, a PRS method that combines GWAS data from both ancestries, to generate a total of 110 PRS for ten diseases. Similar to the simulation results, SBayesRC showed better predictive performance for disease risk than the other methods. Furthermore, the East Asian GWAS data outperformed those from European ancestry for breast cancer, cataract, gastric cancer, and T2D, but neither of the two GWAS ancestries showed a significant advantage on PRS performance for the remaining six diseases. Based on simulation data and real data studies, it is expected that SBayesRC will offer superior performance for East Asian populations, and PRS generated using GWAS from non-East Asian may also yield good results.

## Introduction

Genome-wide association studies (GWAS) have provided information on a large number of genetic variants that contribute to the risk of complex diseases. The genetic susceptibility of individuals to disease can be estimated by calculating the polygenic risk score (PRS) using the associated genetic variants. There has been considerable interest in PRS and the field is growing rapidly, with more than 2700 PRS algorithms presented in the open resource catalog^1^. In addition, evidence for the clinical utility of PRS in diseases such as coronary artery disease (CAD)^2^, breast cancer^3^, and diabetes^4^ is currently increasing^5^, and the possibility of applying PRS for early detection, risk stratification, and personalized treatment of complex diseases has been suggested^6,7^.

PRSs are calculated from the number of alleles of genetic variants, typically weighted by the effect of the variants, estimated from GWAS data. In recent years, various methods for calculating Polygenic Risk Scores (PRS) have been developed. These methods include PRSice^8^, which employs linkage disequilibrium (LD) clumping and *P*-value thresholding (P + T), LDpred^9^, SBayesR^10^, and PRS-CS^11^, which utilize Bayesian regression frameworks; and LDpred-funct^12^ and SBayesRC^13^, which incorporate additional functional annotations. These methods differ in two key criteria: which genetic variants are included in the study, and how to apply weights for genetic variants. Frequently, comparisons between these methods are conducted using simulated data and real-world examples^14^.

Choosing an appropriate GWAS is one of the most important considerations to optimize PRS performance^15^. When selecting a GWAS, the ancestry of the study population is a key factor, since the transferability of PRSs across populations is poor owing to differences in allele frequencies and LD patterns of genetic variants^16,17^. Although the number of GWAS has been increasing in non-European ancestries^18^, most are still performed in European ancestry^19^. This imbalance in GWAS results has led to twice as many PRS studies for European than non-European ancestries^17^. Moreover, the performance of PRS when applying data from GWAS conducted in European ancestry to populations with non-European is unclear.

To explore the performance of PRS for those of non-European ancestry, we tested PRSs under various conditions in a South Korean cohort, Health Examinees (HEXA) of the Korean Genome and Epidemiology Study (KoGES)^20^. We employed five PRS methods based on single GWAS data: lassosum^21^, LDpred-funct, PRSice, PRS-CS, and SBayesRC. The predictive performance of the five PRS methods was assessed using simulated data representing different genetic architectures. In addition, we generated PRSs for ten diseases: asthma, breast cancer, CAD, cataract, gastric cancer, glaucoma, hyperthyroidism, hypothyroidism, osteoporosis, and type 2 diabetes (T2D), using the five PRS methods, and PRS-CSx, which allows for the integration of GWAS data from multiple populations. Biobank-scale GWAS summary statistics from European and East Asian cohorts, UK Biobank (UKB) and BioBank Japan (BBJ)^22,23^ were used, and each PRS method and GWAS population were compared using two predictive performance metrics. Our results can provide guidance in selecting an appropriate PRS method and its corresponding GWAS for a specific population of interest.

## Results

For the analysis, we used data from HEXA, which consists of over the 40-year-old South Korean adults^24^. Table 1 presents the descriptive characteristics of the participants for the 10 diseases: asthma, breast cancer, CAD, cataract, gastric cancer, glaucoma, hyperthyroidism, hypothyroidism, osteoporosis, and T2D. For each disease group, more than 300 cases and 30,000 controls were included, and the average age of disease cases was higher than that of the controls (*P* < 0.05, Student’s t-test). For asthma, hyperthyroidism, hypothyroidism, and osteoporosis, there was a significantly higher proportion of women in the disease cases and these diseases are known to affect women more frequently^25–27^. For T2D, CAD, and gastric cancer, the incidence in men was higher, which is in accordance with previous research^28–30^. In the disease groups for asthma, CAD, and T2D, for which body mass index (BMI) is a risk factor^31–33^, the average BMI was higher than that in the control groups. The SNP-heritability of the diseases in HEXA was varies from 0.08 to 0.48 (Table S1).

**Table 1:** Basic characteristics of Health Examinees participants.

	Case	Control
Asthma		
N	959	56,702
Age, years	55.4 (8.4)	53.8 (8)
Women	682 (71.1%)	37,064 (65.4%)
BMI	24.3 (3.2)	23.9 (2.9)
Breast cancer		
N	351	30,752
Age, years	54 (7.1)	52.9 (7.7)
Women	351 (100%)	30,752 (100%)
BMI	23.5 (2.8)	23.6 (2.9)
CAD		
N	1643	56,022
Age, years	59.9 (6.8)	53.6 (8)
Women	783 (47.7)	36,965 (66%)
BMI	24.9 (2.9)	23.9 (2.9)
Cataract		
N	2068	56,544
Age, years	61.8 (6.3)	53.5 (7.9)
Women	1222 (59.1%)	37,128 (65.7%)
BMI	24.3 (2.8)	23.9 (2.9)
Gastric cancer		
N	302	48,150
Age, years	58.2 (7.9)	53.6 (8)
Women	137 (45.4%)	31,233 (64.9%)
BMI	22.1 (3.1)	23.9 (2.9)
Glaucoma		
N	374	47,028
Age, years	59.6 (7.5)	53.7 (8)
Women	204 (54.5%)	31,125 (66.2%)
BMI	23.9 (2.8)	23.9 (2.9)
Hyperthyroidism		
N	836	38,151
Age, years	54.5 (7.7)	53.7 (8.1)
Women	725 (86.7%)	24,748 (64.9%)
BMI	23.4 (2.8)	23.9 (2.9)
Hypothyroidism		
N	860	38,151
Age, years	54.2 (7.4)	53.7 (8.1)
Women	800 (93%)	24,748 (64.9%)
BMI	23.6 (3)	23.9 (2.9)
Osteoporosis		
N	3010	54,641
Age, years	59.6 (6.4)	53.5 (8)
Women	2878 (95.6%)	34,863 (63.8%)
BMI	23.5 (2.8)	23.9 (2.9)
T2D		
N	4886	51,340
Age, years	57.9 (7.4)	53.4 (8)
Women	2424 (49.6%)	34,416 (67%)
BMI	25 (3.1)	23.8 (2.8)

All data are presented as mean ± standard deviation or numbers (%).

*BMI* body mass index, *CAD* coronary artery disease, *T2D* type 2 diabetes.

### Simulations for evaluating PRS methods in East Asian

We examined the predictive performance of five PRS methods, lassosum, LDpred-funct, PRSice, PRS-CS, and SBayesRC, that utilize single GWAS summary statistics across a range of simulated genetic architectures. We used individual-level genotype inputs from HEXA and applied training and testing sets (Methods). The prediction accuracy for all methods was assessed by calculating Nagelkerke’s R^2^ between the observed and predicted traits in an independent testing set.

**Figure 1:**
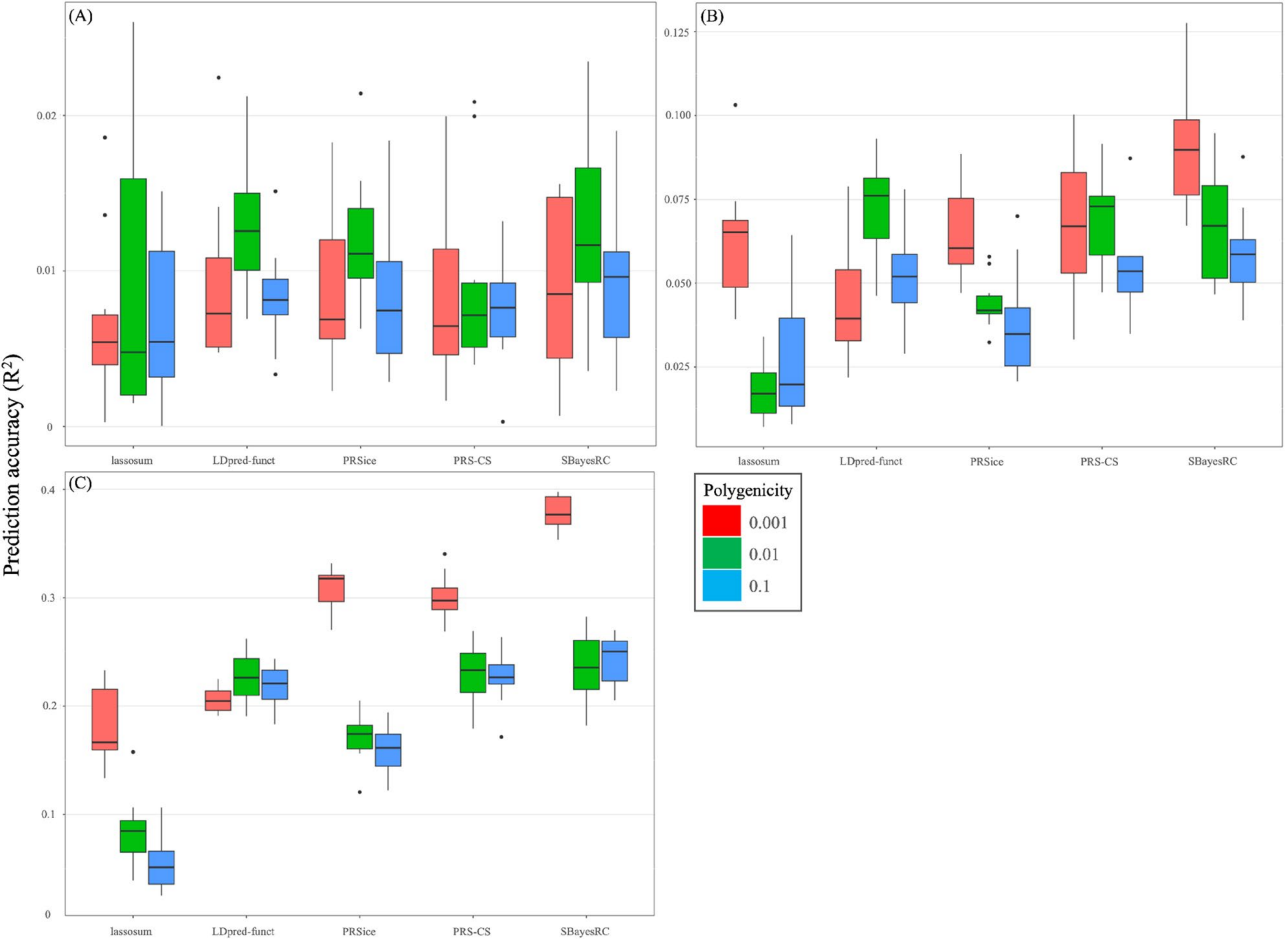
Predictive performance of five polygenic prediction methods in simulation studies. East Asian sample of the 1000 Genomes Project phase 3 was applied as an external linkage disequilibrium (LD) reference panel. Nagelkerke’s R^2^ was used to quantify the prediction accuracy between the predicted and observed traits in a separate test dataset. Each panel correspond to three heritability (0.1 in A, 0.3 in B, and 0.7 in C) and was simulated in three genetic architectures (0.001, 0.01, and 0.1 Polygenicity). In each box, the central mark represents the mean across 10 simulations, while the edges of the box indicate the 25th and 75th percentiles of the data distribution, with outliers plotted individually.

Figure 1 shows the prediction performance of five PRS methods. As expected, the prediction performance increased in all cases as the heritability increased. In fixed heritability 0.1 (Fig. 1A), the prediction accuracy remained relatively stable as the number of causal variants increased, and there was no notable variation in the performance across the different methods. For the higher fixed heritability (0.3 and 0.7), the overall prediction performance generally decreased as the number of causal variants increased (Fig. 1B,C). Furthermore, SBayesRC and PRS-CS outperformed the other methods, and this difference became more pronounced as the heritability increased from 0.3 to 0.7. When the proportion of causal variants was 0.001, SBayesRC demonstrated better performance than PRS-CS. In contrast, lassosum displayed comparatively lower performance throughout the simulation analysis. LDpred-funct exhibited good performance regardless of the heritability when proportion of the causal variants was 0.01.

### Performance of PRS in an East Asian population

We calculated PRSs for ten diseases using GWAS summary statistics obtained from UKB for the European population and BBJ for the East Asian population. Five PRS methods, including lassosum, LDpred-funct, PRSice, PRS-CS, and SBayesRC, were implemented using single GWAS summary statistics. Furthermore, the PRS-CSx was employed to integrate GWAS summary statistics from UKB and BBJ. A total of 110 PRSs, including 11 PRSs for each disease, were generated. The association between each PRS method and the target diseases through logistic regression is summarized in Table S2.

**Figure 2:**
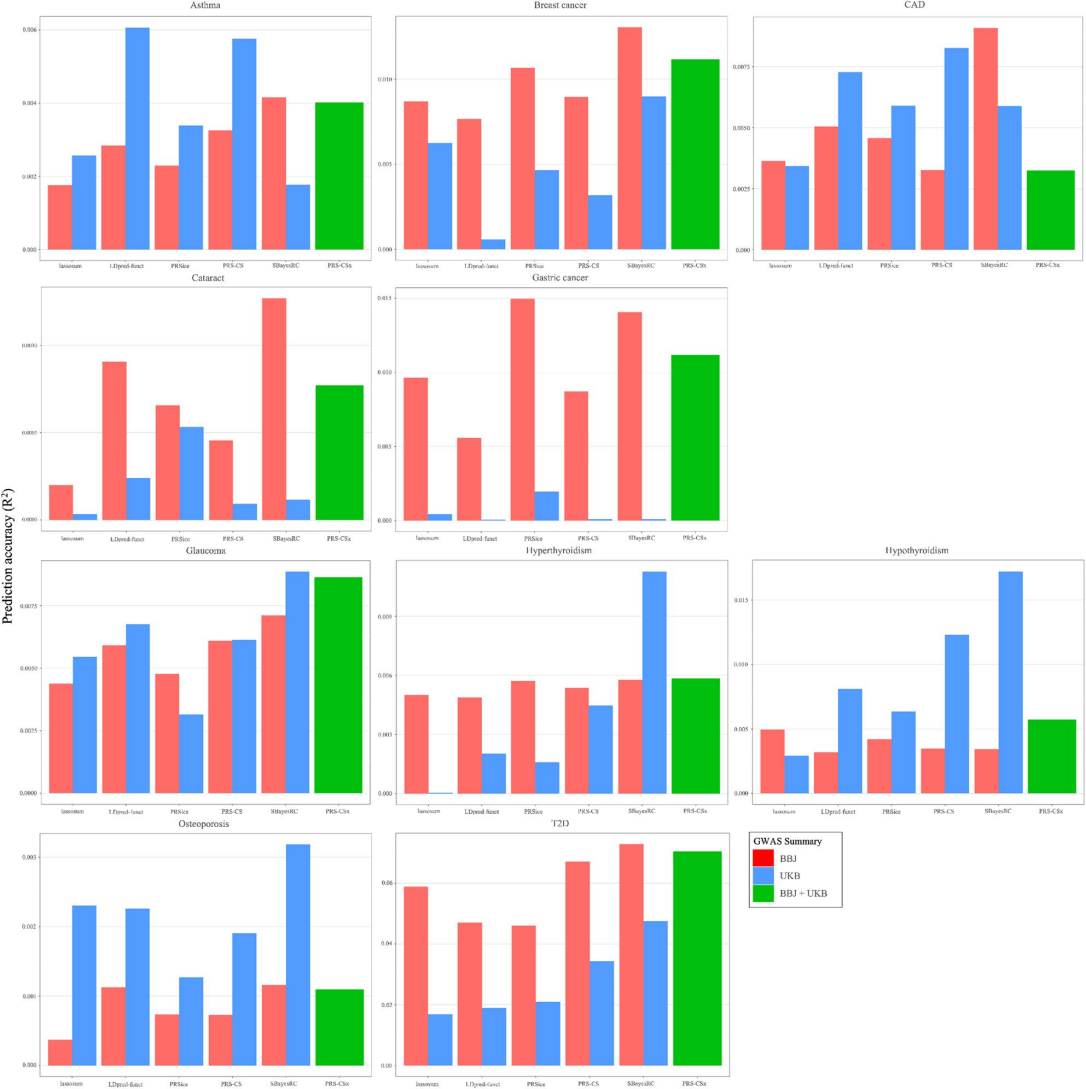
Prediction accuracy estimated as the R^2^ between polygenic risk scores (PRSs) and diseases. Colors of the bar indicate the use of genome-wide association study (GWAS) summary statistic from BioBank Japan (red), GWAS summary statistics from UK Biobank (blue), and the integration of both GWAS summary statistics (green). Six PRS methods were applied to predict ten diseases, asthma, breast cancer, coronary artery disease (CAD), cataract, gastric cancer, glaucoma, hyperthyroidism, hypothyroidism, osteoporosis, and type 2 diabetes (T2D). Prediction accuracy was measured by the Nagelkerke’s R^2^.

To quantify and compare the predictive performance of PRS for each disease, we considered evaluation metrics such as R^2^ and area under the curve (AUC) (Table S3). In the simulated case where only chromosome 1 was considered, we observed that SBayesRC and PRS-CS exhibited the highest prediction accuracy across various genetic architectures. Similarly, SBayesRC performed well for most cases in terms of R^2^ (Fig. 2). When using the summary statistics from BBJ, SBayesRC showed better performance compared to other PRS methods in diseases excluding gastric cancer and hypothyroidism. Additionally, when utilizing the UKB summary statistics, SBayesRC exhibited superior performance in diseases excluding asthma, CAD, cataracts, and gastric cancer. Actually, none of the PRSs utilizing the UKB summary statistics showed significant associations (apply Bonferroni correction < 0.004545) with cataracts and gastric cancer (Table S2). The other four methods, lassosum, LDpred-funct, PRSice, and PRS-CS, did not demonstrate notable performance. PRS-CSx, an extension method of PRS-CS, showed improved performance compared to PRS-CS using UKB and BBJ in breast cancer, cataract, gastric cancer, glaucoma, hyperthyroidism, and T2D. For the remaining diseases, despite utilizing the summary statistics from both UKB and BBJ, PRS-CSx did not exhibit better performance compared to PRS-CS using a single set of summary statistics.

**Figure 3:**
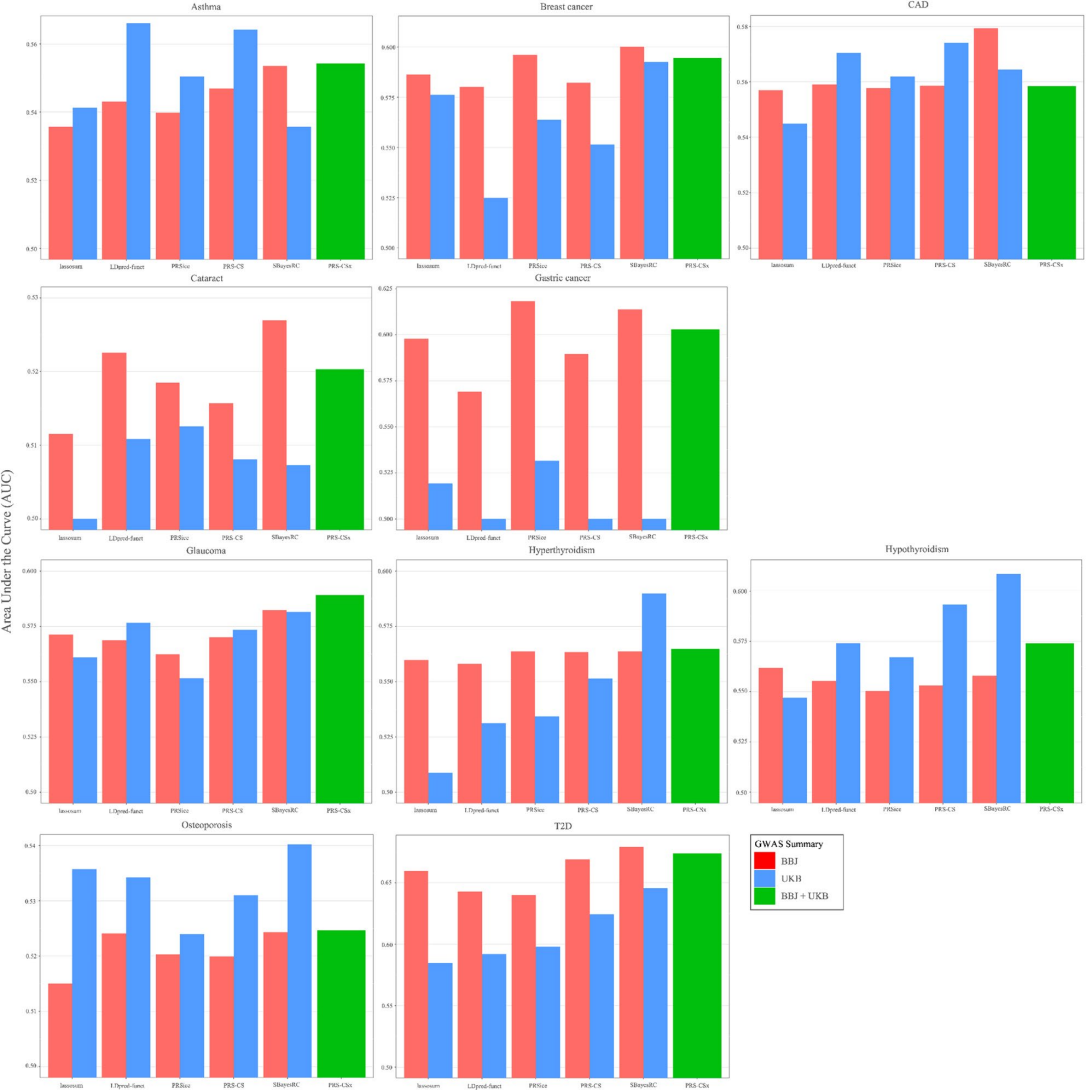
Receiver operator characteristic curves (AUCs) of polygenic risk scores (PRSs) for ten diseases in East Asian individuals. Colors of the bar indicate the use of genome-wide association study (GWAS) summary statistic from BioBank Japan (red), GWAS summary statistics from UK Biobank (blue), and the integration of both GWAS summary statistics (green). Six PRS methods were applied to predict ten diseases, asthma, breast cancer, coronary artery disease (CAD), cataract, gastric cancer, glaucoma, hyperthyroidism, hypothyroidism, osteoporosis, and type 2 diabetes (T2D). The values of AUC were calculated as the average of five-fold cross-validation.

AUC, which estimate the probability that the predicted risk of a randomly selected case is higher than the predicted risk of a randomly selected control, also demonstrated a similar pattern to R^2^ (Fig. 3). In diseases other than gastric cancer and hypothyroidism, the use of summary statistics from the BBJ dataset revealed that SBayesRC exhibited higher AUC compared to other PRS methods. In the case of utilizing UKB summary statistics, SBayesRC demonstrated superior AUC performance in diseases excluding asthma, CAD, cataracts, and gastric cancer as well. PRS-CSx, utilizing both UKB and BBJ summary statistics, demonstrated higher AUC value compared to other PRS methods in cataracts. Furthermore, it showed improved AUC performance compared to the conventional PRS-CS method in diseases excluding asthma, CAD, hypothyroidism, and osteoporosis.

## Discussion

To date, the utility and performance of PRS methods for disease risk prediction have been predominantly investigated in populations with European ancestry. In addition, the transferability of the European PRS to East Asian populations has remained unclear. Given the deficiency of PRS studies for East Asian population, we explored not only which PRS calculation methods proved optimal for specific diseases in an East Asian population, but also whether the PRS generated using GWAS data from European ancestry is effective for risk prediction in East Asian. In the current study, we assessed PRSs for ten diseases in Korean population. Through simulation studies on various genetic architectures, we investigated the performance of five PRS methods (lassosum, LDpred-funct, PRSice, PRS-CS, and SBayesRC) that using a single set of GWAS summary statistics in Koreans. Afterwards, we applied the five PRS methods to the ten diseases using GWAS summary statistics from both East Asian and European ancestries, respectively. Furthermore, we applied PRS-CSx as an algorithm utilizing GWAS summary statistics from multiple populations, resulting in a total of 110 PRS being generated. The performance of each PRS was compared using metrics such as R^2^ and AUC, based on the results from the simulation.

Among the various PRS methods, SBayesRC showed the best performance in simulation data with high fixed heritability of 0.3 and 0.7 (Fig. 1). Consistent with the simulation results, SBayesRC demonstrated the best performance in each GWAS summary data of BBJ and UKB, for diseases other than the three diseases with the lowest heritability (asthma, CAD, and cataract) and gastric cancer (Figs. 2 and 3, Table S1). Furthermore, when applying BBJ summary statistics from the same East Asian ancestry with HEXA-KoGES population, SBayesRC exhibited the best performance even for the three diseases with the lowest heritability and showed the second-highest performance in gastric cancer. Based on the results of both simulation and real data analysis, it is expected that utilizing SBayesRC for East Asian PRS studies with GWAS summary statistics from same ancestry would be yield good performance. Additionally, it is anticipated that SBayesRC would exhibit notable performance for East Asian PRS studies using GWAS summary statistics from other ancestries, including European ancestry.

The transferability of PRSs across populations is hindered by disparities in allele frequencies and LD patterns of genetic variants^17^. In breast cancer, cataract, gastric cancer, and T2D, the BBJ GWAS summary statistics from the same ancestry with HEXA showed better predictive performance compared to the UKB summary statistics (Figs. 2 and 3). For the remaining diseases, the performance of BBJ and UKB was comparable, with some instances showing that UKB had better performance. While complex genetic mechanisms may be involved, the difference in statistical power between the BBJ and UKB GWAS summary statistics could be one possible reason. Examining the diseases where BBJ showed better performance, it can be attributed to the observed SNP-heritability, which was observed to be approximately 2.8 to 10 times higher in BBJ compared to that of UKB (Table S4). In the case of the remaining diseases, the observed SNP-heritability in BBJ was either less than twice as high as UKB or higher in UKB. Similar to previous studies^34,35^, our results highlight an opportunity to use large-scale European GWAS data for the construction of PRSs in East Asia.

In our study, we conducted PRS study in an East Asian cohort using not only GWAS summary data from the same ancestry but also from European ancestry. The study results may be specific to the Korean, since there is limited research on cross-ancestry PRS studies for non-European populations, and Asian populations are known to be ethnically and genetically diverse^36^. Comparing our findings to a study that investigated the transferability of PRS from UKB European to UKB East Asian populations^37^, we observed differences in AUC for most diseases (Fig. S1). In the future, more precise comparisons can be conducted by using the same PRS method and accounting for various covariates.

In summary, we generated PRS for ten diseases in East Asia using GWAS data from European and East Asian ancestries. We employed six PRS calculation methods, including five single GWAS data-based methods and one multi-GWAS data-based method. We estimated the predictive performance of various PRSs using two metrics and showed that a PRS based on GWAS, not only from East Asian but also from European ancestry, works well as a predictor of disease risk in East Asia. Furthermore, through simulation analysis and real data analysis, we showed that SBayesRC exhibited superior performance in the Korean cohort. While it is evident that a grid search, encompassing all known PRS methods and GWAS summary statistics, is the optimal approach to identify the most suitable PRS model, our study results can assist researchers in selecting the appropriate PRS method and GWAS data. Further exploration of diverse PRS methods, various traits, and a wide range of study population is necessary to validate our findings.

## Methods

### Study populations

The present study was conducted using community-based genomic cohort data from the HEXA of the Korean Genome and Epidemiology Study^24^. The survey for the HEXA study was performed at 38 hospitals and local health-screening centers from 2004 to 2013, following standardized procedures. In total, 65,642 urban participants completed the initial and follow-up surveys. Epidemiological data were provided by the Korea Centers for Disease Control and Prevention. For sample quality control, participants with a genotype relative score greater than 0.125 or a body mass index outside the criteria of 15–50 were excluded.

### Genotype data

The genotype data were produced by the Korea BioBank Array, which is optimized for the Korean population and includes 833,535 single nucleotide polymorphisms (SNPs)^38^. Imputation analysis was conducted with ShapeIT v2^39^ and IMPUTE v2^40^ using 1000 Genomes Phase 3 data (1 KG) as a reference panel^41^. For quality control, SNPs with minor allele frequency less than 0.01 or Hardy–Weinberg equilibrium *P*-values less than 10^–6^ or missing data > 0.05 were excluded. A total of 7,915,509 SNPs remained.

### Phenotype definition

For the PRS analysis, we selected the disease based on the following criteria:

1. The number of disease cases ≥ 300 in HEXA.
2. The SNP-heritability of the disease > 0 in HEXA.
3. GWAS summary statistics for the disease are available from both BBJ and UKB.

As a results, ten diseases (asthma, breast cancer, CAD, cataract, gastric cancer, glaucoma, hyperthyroidism, hypothyroidism, and osteoporosis, T2D) passed the criteria.

Participants who constituted the T2D case and control groups were identified by their answers to the questionnaire on T2D diagnostic history and fasting glucose level. Those who replied ‘Yes’ to the questionnaire or had a fasting glucose level above 126 mg/dL were classified into the case group, and those who answered ‘No’ to the questionnaire and had a fasting glucose level less than 126 mg/dL constituted the control group.

We identified case groups for breast cancer and gastric cancer from participants who responded 'yes' to the questionnaire on cancer diagnosis. Among these participants, those who indicated “breast cancer” in response to the question on cancer type were classified as the breast cancer case group, while those who indicated “gastric cancer” were classified as the gastric cancer case group. The cancer control group was defined as those who answered ‘No’ to the questionnaire on cancer diagnosis.

For the other diseases (asthma, CAD, cataract, glaucoma, hyperthyroidism, hypothyroidism, and osteoporosis), the participants were classified using a diagnostic history questionnaire for each disease. Those who answered ‘Yes’ were defined as disease cases, and those who answered ‘No’ were defined as controls. The case group for all the diseases comprised more than 300 individuals. The characteristics of the samples are listed in Table 1.

### PRS calculations

For the PRS calculation, GWAS summary statistics from the UKB and BBJ were selected. We used a total of 20 summary statistics. GWAS summary statistics were obtained from the NHGRI-EBI GWAS Catalog (www.ebi.ac.uk/gwas) and JENGER (jenger.riken.jp/result)^23,42^. The information of GWAS data is shown in Table S5.

We applied five methods for PRS calculation, lassosum^21^, LDpred-funct^12^, PRSice^8^, PRS-CS^11^, and SBayesRC^13^, each using a single GWAS summary statistics. lassosum uses the lasso regression to select informative SNPs, based on their effect sizes. It allows for tuning of parameters without the need for external validation datasets or phenotype data, using a pseudovalidation. We used the ancestry-matched LD reference panel of the 1 KG.

LDpred-funct is a method that leverages trait-specific functional priors. It fits functional priors using a baseline-LD model that includes coding, conserved, regulatory, and LD-related annotations. LDpred-funct estimates the posterior mean causal effect sizes of variants by considering both functional priors and LD between variants. As an input parameter, SNP-heritability was calculated using LDSC^43^.

PRSice, is a P + T method that tests PRS at a large number of thresholds and applies the best-fit PRS to the study samples. PRS is calculated as the sum of the remaining independent SNPs with a GWAS association *P*-value below a threshold *P*_T_. We consider *P*_T_ (minimum = 5E−08, maximum = 0.5, interval = 5E−05) and other parameters (physical distance > 250 kb and r^2^ < 0.1) with default setting. The *P*_T_ value that maximizes the prediction accuracy in the validation dataset is selected, and the performance of the optimized PRS was assessed in an independent testing dataset.

PRS-CS is Bayesian method that leverages GWAS summary statistics and LD information to estimate the effect size of each variant on a trait. It employs a continuous shrinkage prior to SNP effect sizes, which reduces the influence of noisy SNPs and improving the accuracy of the PRS. In the analysis, we used default parameter settings, along with an LD reference panel based on external European and East Asian samples from the 1 KG, considering the ancestry of the GWAS summary statistics.

SBayesRC is a Bayesian method that assumes a multi-normal mixture distribution for SNP effects. It assumes that the effects of standardized SNPs follow a mixture of normal distributions with different variances (0, 0.001, 0.01, 0.1, and 1%), with each SNP explaining genetic variance ranging from zero to 1%. Also, SBayesRC utilizes annotation data that can influence the probability of a SNP being considered causal, as well as the magnitude of its causal effect size. We utilized the provided genomic annotation data and LD reference of EAS and EUR from UKB.

Additionally, we applied PRS-CSx, which an extension of PRS-CS that enables the integration of GWAS summary statistics from various populations. PRS-CSx leverages the correlation among genetic effects while considering the allele frequency and LD information that are unique to each population. We applied the 1 KG LD reference panel in accordance with ancestry.

### Simulations

We performed simulation studies using real genetic data on chromosome 1 of 6000 individuals form the HEXA. Synthetic phenotypes were generated using GCTA^44^ with varying levels of polygenicity (0.001, 0.01, and 0.1), heritability (0.1, 0.3, and 0.7), and a default prevalence of 0.1. The sample was divided into a training set and a test set at a ratio of 4:1. GWAS was performed on the training set, and PRS was calculated for the test set using the five PRS methods. The simulation was repeated 10 times.

### Statistical analysis

SNP quality control, sample filtering, and PRS calculation were performed using PLINKv.1.9.0^45^. GWAS analysis was performed using SAIGE^46^. The SNP-heritability was estimated using LDSC^43^, which utilized pre-calculated LD scores, regression weights, and allele frequencies from the 1 KG in a relevant ancestral population. We excluded variants in the HLA region (hg19, chr6:26 Mb–34 Mb) for the calculation of heritability. For the evaluation of PRS performance, AUC was calculated by applying five-fold cross-validation for each subject of the disease^47^. Student’s t-tests and regression analyses were performed using basic packages of R version 4.05. Nagelkerke’s R^2^ was calculated using R package ‘lrm’. The bar plot and box plot were created using the R package ‘ggplot2’.

### Supplementary Information

Supplementary Information 1.

Supplementary Information 2.

## Data Availability

UKB and BBJ summary statistics used in this study were downloaded from NHGRI-EBI GWAS Catalog (www.ebi.ac.uk/gwas) and JENGER (jenger.riken.jp/result). This paper does not report custom code.
