# Supplementary material for: Evaluation of optimal methods and ancestries for calculating polygenic risk scores in East Asian population
Source: Sci Rep. 2023 Nov 6;13:19195. doi: 10.1038/s41598-023-45859-w (PMC10628155; doi:10.1038/s41598-023-45859-w)
Supplement: Supplementary file 1 — Supplementary Information 1. [file 41598_2023_45859_MOESM1_ESM.docx]

**Evaluation of optimal methods and ancestries for calculating polygenic risk scores in East Asian population**

Dong Jun Kim^1^, Joon Ho Kang^1^, Ji-Woong Kim^1^, Myeong Jae Cheon^1^, Sun bin Kim^1^, Young Kee Lee^1^, Byung-Chul Lee^1*^

^1^Genoplan Korea, Seoul, Korea

*Corresponding author:

Byung-Chul Lee

Genoplan Korea, Seoul, Korea

E-mail: [io@genoplan.com](mailto:io@genoplan.com)

| Table S1. SNP-heritability of ten diseases in HEXA. | | | | | |
| --- | --- | --- | --- | --- | --- |
|  | N cases | | N controls | | *h*^2^ |
| Asthma | 959 | 56,702 | | 5.9% | |
| Breast cancer | 351 | 30,752 | | 10.8% | |
| CAD | 1,643 | 56,022 | | 8.4% | |
| Cataract | 2,068 | 56,544 | | 10.2% | |
| Gastric cancer | 302 | 48,150 | | 25.5% | |
| Glaucoma | 374 | 47,028 | | 48.1% | |
| Hyperthyroidism | 836 | 38,151 | | 11.8% | |
| Hypothyroidism | 860 | 38,151 | | 15.52% | |
| Osteoporosis | 3,010 | 54,641 | | 13.1% | |
| T2D | 4,886 | 51,340 | | 31.4% | |
| h^2^; heritability in a liability scale. CAD coronary artery disease, T2D type 2 diabetes. | | | | | |

| Table S4. SNP-heritability of ten diseases from BBJ and UKB. | | | |
| --- | --- | --- | --- |
|  | *h*^2^ | | |
|  | UKB | BBJ | |
| Asthma | 4.80% | | 2.41% |
| Breast cancer | 1.00% | | 3.11% |
| CAD | 9.06% | | 7.24% |
| Cataract | 0.18% | | 1.17% |
| Gastric cancer | 0.17% | | 1.73% |
| Glaucoma | 0.95% | | 1.79% |
| Hyperthyroidism | 0.61% | | 1.23% |
| Hypothyroidism | 4.60% | | 0.32% |
| Osteoporosis | 1.26% | | 0.71% |
| T2D | 4.29% | | 11.97% |
| h^2^; heritability in an observed scale. CAD coronary artery disease, T2D type 2 diabetes. | | | |

| Table S5. Information of GWAS summary statistics from UK Biobank and BioBank Japan. | | | | | | |
| --- | --- | --- | --- | --- | --- | --- |
|  | UK Biobank | N cases | N controls | Bio Bank Japan | N cases | N controls |
| Asthma | GCST90038616 | 56,087 | 428,511 | JENGER ID: 30 | 8,216 | 201,592 |
| Breast Cancer | GCST90041886 | 10,152 | 446,124 | JENGER ID: 102 | 5,552 | 89,731 |
| CAD | GCST005194 | 34,541 | 261,984 | JENGER ID: 103 | 29,319 | 183,134 |
| Cataract | GCST90038649 | 7,038 | 477,560 | GCST90018594 | 38,194 | 140,532 |
| Gastric Cancer | GCST90043846 | 192 | 456,156 | GCST90018629 | 7,921 | 159,201 |
| Glaucoma | GCST90038648 | 5,126 | 479,472 | GCST90018632 | 8,448 | 168,903 |
| Hyperthyroidism | GCST90038636 | 3,731 | 480,867 | GCST90018640 | 994 | 172,656 |
| Hypothyroidism | GCST90038637 | 23,497 | 461,101 | GCST90018642 | 1,114 | 172,656 |
| Osteoporosis | GCST90038656 | 7,751 | 476,847 | JENGER ID: 79 | 7,788 | 204,665 |
| T2D | GCST90029024 | 19,292^a^ | 440,032^a^ | JENGER ID: 14 | 36,614 | 155,150 |
| CAD coronary artery disease, T2D type 2 diabetes. | | | | | | |
| a. estimated number from 0.42 Case fraction of 459,324. | | | | | | |


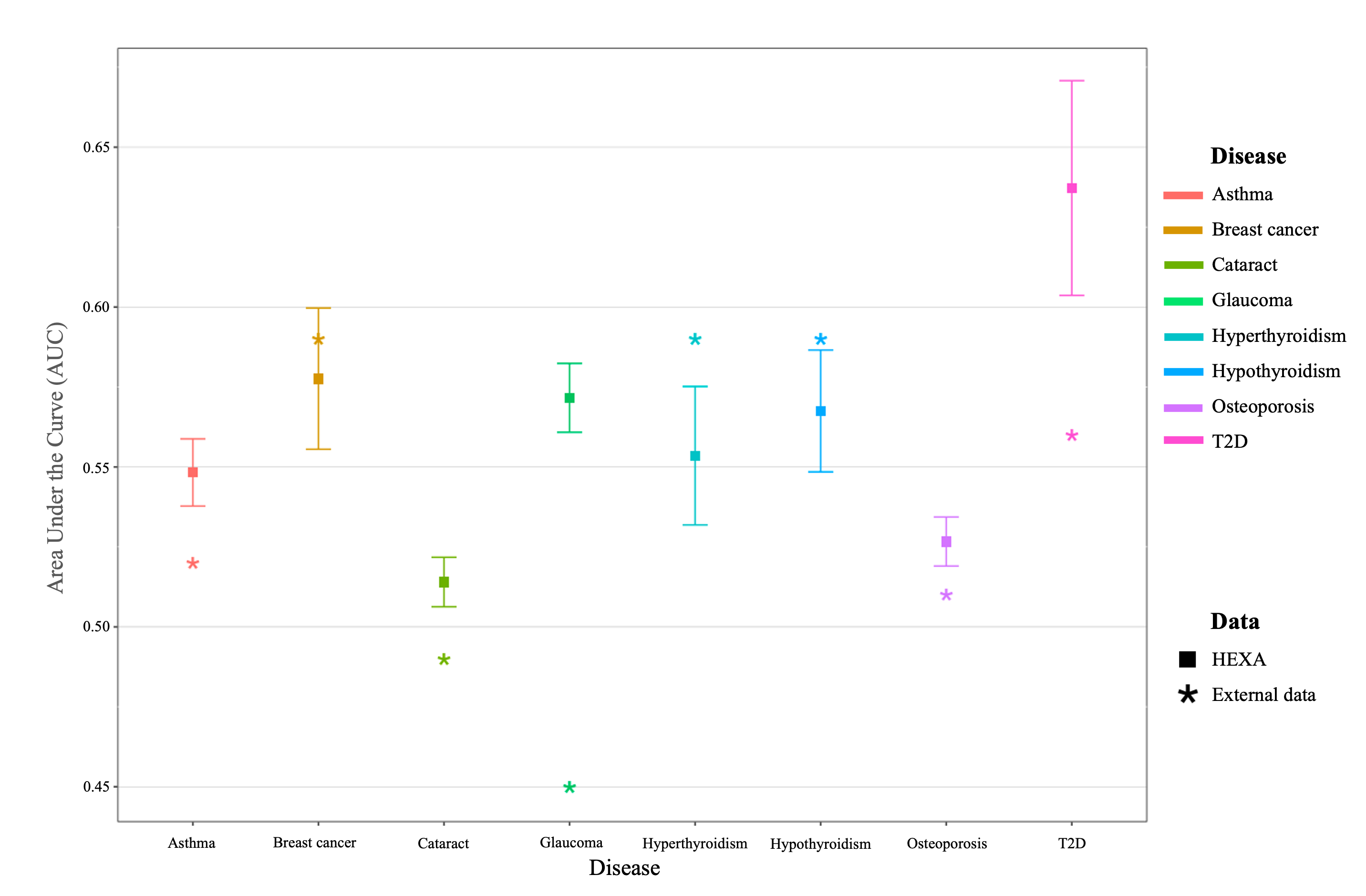


**Figure S1. Prediction performance of diseases in East Asian ancestry**. In each box, the central square mark represents the mean five PRS methods, while the edges of the box indicate the 25th and 75th percentiles of the data distribution. The PRS performance from the external paper is denoted by asterisks (*).
